# Social determinants and BCG efficacy: a call for a socio-biological approach to TB prevention

**DOI:** 10.12688/f1000research.14085.1

**Published:** 2018-02-23

**Authors:** Jennifer B. Dowd, Helen A Fletcher, Delia Boccia

**Affiliations:** 1 Department of Global Health and Social Medicine, King’s College London, London, UK; 2 CUNY Graduate School of Public Health and Health Policy, New York, NY, USA; 3 Department of Immunology and Infection, London School of Hygiene and Tropical Medicine, London, UK; 4 Faculty of Epidemiology and Population Health, London School of Hygiene and Tropical Medicine, London, UK

**Keywords:** tuberculosis, BCG, social determinants, social protection, psychosocial, immunity, vaccine, psychosocial stress

## Abstract

A high burden of TB mortality persists despite the long-term availability of the bacillus Calmette-Guérin (BCG) vaccine, whose efficacy has been highly variable across populations. Innovative and alternative approaches to TB prevention are urgently needed while optimal biomedical tools continue to be developed. We call for new interdisciplinary collaborations to expand and integrate our understanding of how social determinants influence the biological processes that lead to TB disease, how this translates into differential BCG efficacy and, ultimately, how social protection interventions can play a role in reducing the global burden of TB. After providing an overview of the immune pathways important for the establishment of a response to the BCG vaccine, we outline how social determinants and psychosocial stressors can contribute to the observed variation in BCG efficacy above and beyond these biological factors. We conclude by proposing a new interdisciplinary research model based on the integration of social epidemiology theories with biomedical knowledge.

## Introduction

Improvements in the prevention of Tuberculosis (TB) remain an urgent global public health priority, with the disease killing an estimated 1.5 million people annually ^1^. This high burden of mortality persists despite the long-term availability of the bacillus Calmette-Guérin (BCG) vaccine, whose efficacy has been highly variable across populations. Recent trials of the vaccine candidate MVA85A, designed to boost BCG efficacy, showed high levels of immunogenicity in UK adults but poor levels in South African infants, highlighting a frustrating but common inconsistency of responses across populations ^2, 3^. This inconsistency of response is not limited to TB vaccines but is also observed in malaria and HIV vaccine development, where vaccine candidates often appear highly effective in early phase trials and yet perform below expectation in efficacy trials ^4^. Calls to solve the puzzle of heterogeneous response to TB vaccination have traditionally focused on the technology of vaccine design and the interaction of *Mycobacterium tuberculosis (M.tb)* with the immune system at the molecular level ^5, 6^. These are crucial areas of investigation, but we argue that such large gaps in translation from the laboratory to human populations require new approaches to understand how social and biological variables interact to shape immune response and vaccine efficacy.

While research on both the social and biological determinants of TB risk is well developed, thus far the two lines of enquiry have proceeded largely independently. The social epidemiology of TB typically examines social determinants of risk for exposure, diagnosis, and treatment, such as malnutrition, poor ventilation and overcrowding, and barriers in access to health care ^7^. The more limited literature on social factors and TB vaccination focuses on factors such as vaccine distribution and uptake, but not immune response or vaccine efficacy ^8^, and does not attempt to understand how social determinants may influence the expression of biological markers relevant to BCG efficacy and risk of TB disease. To date, variability in BCG efficacy remains explained in terms of interference from previous exposure to tuberculous or non-tuberculous mycobacteria ^6^. Meanwhile outside of the context of TB, psychosocial and social influences on host immunity have been well described ^9– 11^, but research on socio-environmental factors and the biology of TB vaccine response is lacking.

In this paper, after providing an overview of the immune pathways important for the establishment of a response to the BCG vaccine, we propose a new focus on social determinants and psychosocial stressors that can contribute to the observed variation in BCG efficacy above and beyond these biological factors. We conclude by proposing a new interdisciplinary research model based on the integration of social epidemiology theories with biomedical knowledge.

## BCG immune response and vaccine efficacy

While TB disease is a classic example of a disease of poverty, it is ultimately the failure of the immune system to contain infection which leads to active disease. Although we do not have a validated immune correlate of BCG vaccine efficacy, we know from studies of host-genetic susceptibility that cellular immunity plays a critical role in protection from TB disease ^12^. Defects in the IFN-γ and IL-12 pathways, T cell and NK cell defects (GATA2 deficiency) and defects in monocytes and dendritic cells (IRF8 deficiency and CGD) are associated with susceptibility to mycobacterial disease ^12^. Vaccination with BCG leads to the expansion of both classical antigen specific CD4+ and CD8+ T cells and non-classical cells such as CD1 and HLA-E restricted T cells ^13^. There is also an NK response following immunization with BCG, likely driven by IL-2 secretion from mycobacterial antigen specific CD4+ T cells ^14^. BCG can modify the innate immune response through induction of epigenetic changes in monocytes and dendritic cells ^15^. A role for BCG vaccine induced IFN-γ in protection from TB disease has been suggested in an infant study where both BCG-specific IFN-γ secretion and Ag85A specific IgG were associated with lower risk of developing TB disease ^14^. The BCG vaccine can therefore interact and modify all the key immune cells and pathways identified in human genetic studies to be critical for protection from TB disease. 

Recently, specific correlates of immune risk for TB disease have been identified in BCG vaccinated infants aged 10 weeks ^16^ and 6 months ^14^ enrolled in TB vaccine efficacy trials in the Western Cape of South Africa. Immune markers associated with TB risk include CD4+ T cell activation ^14^, increased monocyte to lymphocyte ratio ^16, 17^ CMV positivity on ELISPOT ^18^, and Type I Interferon ^19^. These immune correlates highlight a role for the host immune environment in TB disease risk. Pre-vaccination inflammation has been associated with lower efficacy of hepatitis B, yellow fever and HIV vaccines ^20– 22^. Inflammation has also been associated with altered CD4 T cell response following immunization with BCG ^23^. Known environmental drivers of T cell activation include viral infection ^24, 25^, previous exposure to mycobacteria, age and proximity to the equator. However, there are also well-documented social and psychosocial determinants of these immune biomarkers in other contexts, which we describe below.

## The role of socioeconomic and psychosocial factors on immunity

Socioeconomically disadvantaged populations face both material and psychosocial threats to adequate host immunity ^9– 11^. Stress is defined as an event or environmental demand that exceeds an individual’s perceived ability to cope, eliciting physiological stress responses from the hypothalamic-pituitary-adrenal (HPA) axis and the autonomic nervous system to deal with the threat ^26^. There is abundant evidence from developed countries that chronic stressors and negative emotions such as anxiety and depression directly influence the immune system, including greater susceptibility to viral and bacterial infection and inhibited response to vaccines ^9, 27, 28^. These stressors have also been found to be associated with other biomarkers of immunity and inflammation including TNFA, IL6 and IL1B, decreased NK cell function, as well as impaired control over latent viral infections ^9, 11, 29– 31^. Increased monocyte counts and monocyte to lymphocyte ratios have also been found in individuals with depressive symptoms, a common consequence of chronic stress and social deprivation ^32, 33^. Importantly for vaccination of infants, prenatal maternal stress has been shown to influence postnatal immunity in offspring, though this literature is currently more developed in animals than in humans ^34, 35^. Despite the high burden of deprivation and psychosocial stressors in areas where TB is endemic, the links between these exposures and biological immune response to BCG vaccine has not been explored. **

These important but seldom overlapping literatures present a unique opportunity to elucidate how the social environment contributes biologically to variability in BCG vaccine efficacy. As an example of potential insights from such intersection, infection and immune response to one latent herpesvirus, cytomegalovirus (CMV), has been associated with both socioeconomic status and stress in developed countries ^11, 36^. Recent work suggests that CD4+ T cell activation is associated with risk of TB in BCG vaccinated infants in South Africa, with CMV infection emerging as one important driver of this T-cell activation ^14^. These associations suggest a testable pathway from socioeconomic status and maternal stress, CMV reactivation, and impaired immune response to BCG vaccine in infants ^37, 38^. 

## New opportunities for TB prevention: a “social technology” approach

From the pathway above, it could be argued that poverty reduction strategies, such as social protection, directly impacting socioeconomic status and maternal mental health could potentially play a role in increasing BCG efficacy. Social protection has been defined as a range of policies that enable people to cope with and recover from shocks, with the objective of moving people out of extreme poverty and interrupting the transgenerational transmission of inequalities ^39^. Social protection includes widely used poverty-reduction strategies in both low and middle-income countries and encompasses both social security and social assistance interventions. In the latter group, cash transfer interventions are currently the most popular form of social protection ^40^. These typically consist of the provision of regular, non-contributory, monetary benefits to households living in poverty and extreme poverty. They can be given unconditionally or conditionally on a number of educational (i.e. children school enrollment and attendance) and health behaviour requirements (i.e. access to maternal and child care services) that beneficiaries have to meet in order to remain enrolled. Today it is well acknowledged that cash transfers can improve beneficiaries’ socioeconomic status ^41^. More recent literature suggests that cash transfers can significantly improve maternal mental health ^42, 43^ as well as self-perceived happiness among beneficiaries ^44, 45^, pointing to potential benefits on biological markers of stress and immunity.

Based on these promising links, we call for the establishment of an interdisciplinary partnership, preliminarily named the “Social Technology Lab”, aiming to address the knowledge gaps in the pathways linking social protection, BCG immunological response, and TB disease. The ultimate goal of this research effort is the development and testing of a new vaccine R&D model based on the combination of innovative biotechnology tools with poverty reduction strategies, using BCG as proof of concept. We argue that such an innovative paradigm can provide crucial insights into how social factors manifest biologically via immune response to TB vaccine and potentially help explain the puzzle of wide variation in responses. Ultimately, this interdisciplinary approach will increase our understanding of how social determinants influence host immunity and TB risk over the life course, informing the integration of upstream structural and downstream biomedical intervention strategies. 

## Conclusions

A more effective TB vaccine is considered the real game changer in the fight against the disease, however the development and delivery of new products may take decades even with considerable scientific and financial investment. Recent simulations suggest that if existing biomedical tools, including current BCG, were combined synergistically with social protection interventions, this could result in a significant acceleration in the decline of TB incidence globally ^46^. Innovative and alternative approaches to TB prevention are urgently needed while optimal biomedical tools continue to be developed. An interdisciplinary model that expands and integrates our understanding of how social determinants influence the biological processes that lead to TB disease, how this translates into differential BCG efficacy and, ultimately, how this influence can be affected by social protection interventions holds promise for reducing the global burden of TB disease.
