# Supplementary material for: Social determinants and BCG efficacy: a call for a socio-biological approach to TB prevention
Source: F1000Res. 2018 Feb 23;7:224. [Version 1] doi: 10.12688/f1000research.14085.1 (PMC5974585; doi:10.12688/f1000research.14085.1)
Supplement: Peer review history

# Peer review: Referee response for version 1

**DOI:** 10.5256/f1000research.15321.r33031

**Published:** 2018-05-29

**Authors:** Marie-Claude Rousseau

**Affiliations:** 1 Epidemiology and Biostatistics Unit, INRS-Institut Armand-Frappier, Université du Québec, Laval, QC, Canada

In this opinion article, Dowd et al. propose a new approach for tuberculosis (TB) prevention, based on BCG vaccination and poverty reduction strategies. The article is well written, logically organized, and scientifically sound. After reviewing the evidence on immune pathways involved in protection from TB, they describe host factors associated with vaccine efficacy, and address socioeconomic and psychosocial factors affecting immunity. They argue convincingly that there exist several research gaps about the pathways connecting social factors, the immune response elicited by the BCG vaccine, and TB prevention. They recommend the establishment of a new “Social Technology Lab”, an interdisciplinary partnership aiming to address these knowledge gaps. This is a promising and innovative proposal in the context of the worldwide burden of TB and obstacles faced toward designing a more efficacious TB vaccine.

 The authors’ rationale rests on the highly variable efficacy of BCG, as observed across several populations. They provide credible evidence that differences in vaccine efficacy may very well be related to varying socioeconomic and psychosocial factors across study populations. They also document that the host immune environment can modulate TB risk and thus modify the effect of the BCG vaccine. One aspect that seems to be missing from this conceptual framework is that vaccine characteristics are likely related to vaccine efficacy. Indeed, some authors have reported that BCG substrain and route of vaccination influenced BCG virulence and efficacy ^1^^,^^2^^,^^3^^,^^4^. The article would be strengthened by including a few sentences describing the state of knowledge on vaccine characteristics and how they relate to efficacy. This could be integrated in the section “BCG immune response and vaccine efficacy”. Although it is a minor issue for the current article, this aspect is relevant for the future activities of the Social Technology Lab and should be integrated in its investigations.

I have read this submission. I believe that I have an appropriate level of expertise to confirm that it is of an acceptable scientific standard.

## References

[1] BroschRGordonSVGarnierTEiglmeierKFriguiWValentiPDos SantosSDuthoySLacroixCGarcia-PelayoCInwaldJKGolbyPGarciaJNHewinsonRGBehrMAQuailMAChurcherCBarrellBGParkhillJColeST: Genome plasticity of BCG and impact on vaccine efficacy. .2007;104(13) : 10.1073/pnas.0700869104 5596-601 10.1073/pnas.0700869104 17372194PMC1838518

[2] AbdallahAMHill-CawthorneGAOttoTDCollFGuerra-AssunçãoJAGaoGNaeemRAnsariHMalasTBAdroubSAVerboomTUmmelsRZhangHPanigrahiAKMcNerneyRBroschRClarkTGBehrMABitterWPainA: Genomic expression catalogue of a global collection of BCG vaccine strains show evidence for highly diverged metabolic and cell-wall adaptations. .2015;5: 10.1038/srep15443 15443 10.1038/srep15443 26487098PMC4614345

[3] MolivaJITurnerJTorrellesJB: Prospects in Mycobacterium bovis Bacille Calmette et Guérin (BCG) vaccine diversity and delivery: why does BCG fail to protect against tuberculosis?. .2015;33(39) : 10.1016/j.vaccine.2015.08.033 5035-41 10.1016/j.vaccine.2015.08.033 26319069PMC4577463

[4] ZhangLRuHWChenFZJinCYSunRFFanXYGuoMMaiJTXuWXLinQXLiuJ: Variable Virulence and Efficacy of BCG Vaccine Strains in Mice and Correlation With Genome Polymorphisms. .2016;24(2) : 10.1038/mt.2015.216 398-405 10.1038/mt.2015.216 26643797PMC4817822

# Peer review: Referee response for version 1

**DOI:** 10.5256/f1000research.15321.r31152

**Published:** 2018-03-21

**Authors:** Aric A. Prather

**Affiliations:** 1 Department of Psychiatry, University of California, San Francisco (UCSF), San Francisco, CA, USA

There is no question that tuberculosis prevention serves as a pressing global health priority, and while the BCG vaccine is moderately effective, the variability in response among those inoculated raises important questions about what accounts for such variation. Dowd and colleagues make a strong case for the need to investigate more systematically the role of social determinants and psychosocial stressors in predicting BCG response. The call for a “Social Technology Lab” would fill a knowledge gap around how and when social determinants affect vaccination efficacy; however, it raises questions about at what level of measurement one would need to employ to get meaningful answers. Population scientists are often hampered by space constraints around questionnaires to obtain the necessary contextual information to understand psychological constructs, including psychological stress, and there is a need for a common vernacular when discussing stress (see Epel et al., in press ^1^). Similar harmonization around other relevant social factors likely tied to vaccination response is needed. Nevertheless, an interdisciplinary approach to understanding BCG efficacy is well warranted.

 There are a couple of minor concerns that, if addressed, could improve the paper. In paragraph 5, it would be helpful to specify the direction in which immune markers are associated with TB risk. In the following paragraph, the definition of stress could be more specific. In the context of psychosocial factors, stress occurs when one’s perceived demands exceed one’s ability to cope (Lazarus & Folkman, 1984), which often results in a coordinated physiologic and behavioral response to adaptively cope with the perceived demands. Stress as an event, i.e., stressor, is relevant but presumably the stress-immune link of interest in psychological in nature.

I have read this submission. I believe that I have an appropriate level of expertise to confirm that it is of an acceptable scientific standard.

## References

[1] EpelESCrosswellAMayerSEPratherAASlavichGMPutermanEBerry MendesW: More than a feeling: A unified view of stress measurement for population science (in press). .10.1016/j.yfrne.2018.03.001PMC634550529551356
